# The Experiences of Home Care Nurses in Regard to the Care of Vulnerable Populations: A Qualitative Study

**DOI:** 10.3390/healthcare10010021

**Published:** 2021-12-23

**Authors:** Isabel María Fernández-Medina, María Dolores Ruíz-Fernández, Felisa Gálvez-Ramírez, Evangelina Martínez-Mengíbar, Manuel Eduardo Ruíz-García, María del Mar Jiménez-Lasserrotte, Ángela María Ortega-Galán, José Manuel Hernández-Padilla

**Affiliations:** 1 Department of Nursing, Physiotherapy and Medicine, University of Almería, 04120 Almeria, Spain; isabel_medina@ual.es (I.M.F.-M.); mjl095@ual.es (M.d.M.J.-L.); j.hernandez-padilla@ual.es (J.M.H.-P.); 2 Facultad Ciencias de la Salud, Universidad Autónoma de Chile, Temuco 7500000, Chile; 3 Basic Health Zone Puerto de la Torre, Sanitary Distric Málaga-Guadalhorce, Andalusian Health System, 29009 Malaga, Spain; felisa.galvez.sspa@juntadeandalucia.es; 4 Basic Health Zone Bajo Andarax, Almeria Distric, Andalusian Health System, 04009 Almeria, Spain; evangeli@ual.es; 5 San Cecilio Hospital, Andalusian Health System, 18016 Granada, Spain; meduardo.ruiz.sspa@juntadeandalucia.es; 6 Department of Nursing, University of Huelva, 21007 Huelva, Spain; angela.ortega@denf.uhu.es; 7 Department of Adult, Child and Midwifery, School of Health and Education, Middlesex University, London NW4 4BT, UK

**Keywords:** home care, vulnerable patient, home care nurses, experience, qualitative research

## Abstract

Home care nurses have become the main references in home care for vulnerable patients. In patients’ homes they offer comprehensive and continuous care to both the vulnerable population and their families. The aim of this qualitative study was to explore experiences and perspectives of home care nurses regarding the care of vulnerable patients in Spain. We conducted in-depth semi-structured interviews with 15 home care nurses working with a vulnerable population. From a data analysis, two themes and four subthemes emerged: (1) “barriers to providing home care to vulnerable populations”, with the following subthemes: “the particularities of the patient and their home caregivers” and “perceived barriers for the involvement of home care nurses in the care”; and (2) “the emotional cost of home care” with the subthemes “home care is draining for caregivers” and “the impact of home care on the home care nurses”. These findings show us that nurses face a number of difficulties in home care for vulnerable patients. The training of nurses in certain competencies and skills by the social health services would enhance the quality of care offered to these patients.

## 1. Introduction

Primary health care in Spain provides the services of prevention and promotion of health, acute and chronic care, home care and community care activities [1]. Home care is defined as the provision of health services by formal or informal caregivers at home in order to promote, restore, or maintain the maximum level of comfort, functionality and health [1,2]. In recent decades, sociodemographic changes have increased the demand for home care [3]. The ageing of the population, the shortage of family caregivers, and the offering of care to complex patients at home have made home care nurses a benchmark in this care model [4,5]. The home care offered covers a population of all ages and in different social and health circumstances [6]. Family caregivers are also a valuable resource in home care and should be considered on an equal basis with patients [7].

The home is the ideal place to detect, assess, support, and control the health problems of both the individual and the family, favoring autonomy, empowerment and the improvement of people’s quality of life [8]. Home care nurses thus face people or families in situations of vulnerability or susceptibility, that is, those who are more prone to developing health problems, either due to the physical, genetic, psychological or social condition in which they are immersed or because they are exposed to certain external environmental factors [9,10]. Among the latter we can highlight the access and quality of health services, the deficit of social support, social marginalization or their socioeconomic level [11]. This vulnerable population group includes people with multiple diseases, the elderly, victims of violence or abuse, congenital diseases, disabilities, mental health problems or addictions [12].

Optimal home care for this type of population will depend on the collaboration of the family and the performance of multidisciplinary teams [13]. Home care nurses assume the continuity of care at home and lead the coordination between different professionals and care settings [14]. Thus, considering home care nurses’ role in the care of vulnerable populations, it is meaningful to explore their experiences in order to improve the care provided and adjust interventions according to the patients and caregivers’ needs. Therefore, this study purpose was to explore the experiences and views of home care nurses on the care of vulnerable patients in Spain.

## 2. Methods

### 2.1. Design

A qualitative research approach with a descriptive design was used to explore the home care nurses’ experiences. A qualitative descriptive methodology provides an in-depth understanding of a particular phenomenon and allows us to explore important healthcare questions that have a direct impact on nursing practice. In the construction of the interpretive descriptive studies, the researcher should embrace his or her own disciplinary orientation [15]. Prior to the study, the researchers had had extensive knowledge and experience as home care nurses in the care of vulnerable patients.

### 2.2. Study Setting and Participants

Participants were recruited using a purposeful sampling technique as a way to identity participants with considerable experience of the phenomenon. The researchers invited home care nurses from various healthcare centers in southern Spain to take part. Home care nurses received an email with the study information, and those who were interested in participating in the study contacted the first author to schedule an in-person meeting. The inclusion criteria were: having at least 5 years’ continuous experience as a home care nurse, and agreeing to participate in the study. A total of 40 home care nurses from different health care centers were invited, but only 15 (eight women, seven men) accepted to participate in the study. No participant dropped out of the study. The mean age of the participants was 49 years (SD 10.81), and they had worked as home care nurses for a mean of 21.06 years (SD 12.30). The sociodemographic characteristics of the participants are shown in Table 1.

### 2.3. Data Collection

Data were collected between September and December 2020 using semi-structured interviews. We used an interview guide elaborated by the researchers for this study based on researchers’ previous clinical experience and evidence review. The interview guide was according to the purpose of this study and included a series of open-ended questions belonging to different aspects of home care for vulnerable populations (Table 2). Semi-structured interviews began with socio-demographic information and an introductory question about their experiences in home care and requirements and barriers for implementing home care for vulnerable patients. The interviews were conducted face-to-face by the first author and took place in a private meeting room at the local university. Each participant was only interviewed once, and interviews lasted an average of 40 min. All interviews were audio-recorded, and the interviewer took notes that helped to modify some questions initially not included in the interview protocol. During the process, the researchers took notes. Data collection was stopped when data saturation was achieved, that is, no new information or themes arose in the empirical data.

### 2.4. Data Analysis

The audio-recorded interviews were transcribed verbatim by the last author. Significant fragments of the text close to the research question were selected as citations, organized and coded according to their content. Numerous memos were written to identify meaningful segments of data and their relationships during the coding process. In the interpretative phase of the analysis, researchers established the patterns of similarities and differences within the data [15]. The first and last author grouped significant fragments of data into sub-themes and themes based on their similarities. A table with examples of significant quotes, units of meaning, subthemes, and themes was developed (Table 3). Finally, this analysis was verified by the second author. A computer-assisted qualitative data analysis program, ATLAS.ti version 9.0 (Scientific Software Development GmbH, Berlin, Germany), was used to facilitate the understanding of meaningful fragments, organize and code the data.

### 2.5. Rigour

The rigor of the study was assured through the criteria of credibility, confirmability, dependability, and transferability (Lincoln and Guba, 1985). To confirm credibility, all participants received a copy of their transcript in order to confirm the answers. Interviews were conducted by researchers who had no relationship with the participants in order to demonstrate confirmability. To assure dependability, the results of the data collection and the interpretation of the data were carried out by two researchers, and similarities and differences were discussed. Furthermore, a detailed description about data collection and the steps of the research was provided. Finally, transferability was supported by checking data saturation throughout participants’ narratives. In addition, the consolidating criteria for reporting qualitative research (COREQ) checklist was followed to ensure the quality of the study [16].

### 2.6. Ethical Considerations

The study was approved by the Ethics and Research Committee of the Department of Nursing, Physiotherapy and Medicine at the University of Almería (EFM-64/20). The participants received verbal and written information about the aim of the study prior to collecting any data. Their participation was voluntary, and an informed consent was obtained prior to audio recording. The anonymity of the participants and the confidentiality of the data were guaranteed throughout the data analysis.

## 3. Results

Two themes and four subthemes emerged from the analysis of the data. All of them help us to understand how home care nurses perceive the care of vulnerable patients at home. Furthermore, in the description of results, a selection of the most representative quotes is provided.

### 3.1. Theme 1: Barriers to Providing Home Care for Vulnerable Populations

The home care of the vulnerable patient depends on the close collaboration between the patient, family and home care nurses, as well as a good health system provider of resources. This theme reveals the barriers perceived by our participants that hinder home care for vulnerable patients.

#### 3.1.1. Subtheme 1.1: The Particularities of the Patient and Their Home Caregivers

Our participants stressed that communication and dialogue between home care nurses and the vulnerable patient and their family and/or caregivers is essential to establish in the first instance a nursing care plan at home. One of the priorities of the home care nurses in our study was the empowerment of the patient and their family in complying with the therapeutic regime and in making decisions regarding health problems. The empowerment of the patient and their caregiver encourages self-care, independence and a reduction in the need for assistance from others in the opinion of our participants. However, most of our participants pointed out that the sociocultural level of the patient and their caregivers makes it difficult to comply with the recommendations given, and, therefore, home care.

*“There are patients or relatives who do not have sufficient knowledge or are not capable of understanding the recommendations and carrying them out … therefore one of the aims is to ensure that people understand the recommendations and treatment in order to achieve therapeutic adherence, this being fundamental so that their health problems do not get worse … do not get out of balance”.*

(Participant 5)

Another factor that, according to the home care nurses, hinders home care for vulnerable patients is the absence or scarcity of a family and/or social support network. In these cases, the work of the nurses was focused on assessing the patient’s problem and seeking the resources and support necessary to solve it.

*“It is true that I find difficulty with patients who live alone … vulnerable patients who would need someone and resources … A patient who needs help for self-care, connected to oxygen, who need medication and only has home help for two hours … really, that patient cannot handle their situation well, because they lack support”.*

(Participant 12)

From the narratives of our participants, it can be deduced that informal care in the home of the vulnerable patient is a natural responsibility taken on by women as their duty within the family nucleus. However, the home care nurses in our study believe that home care for vulnerable patients is an arduous task that should be shared between all members of the family nucleus.

*“It is incredible that when we get to the homes, the woman is still appointed to do this task (care)… in order not to generate conflicts in the family, women prefer to take on this role with all the consequences … but care is not something hereditary, care must be shared because the tasks are difficult”.*

(Participant 7)

However, according to some home care nurses, the existence of several caregivers with different points of view is a source of conflict that makes it difficult to provide home care. Faced with the resolution of family conflicts, the home care nurses adopted the role of “patient advocate of the patient”, relegating informal care at home to the caregiver with the greatest involvement.

*“The problem arises when there are several people who take care of someone, and each thinks in a way and has an interest, and you, who do you go along with? … and in the end you support the one you see who cares best and most about the patient… instead of the one who cares more about him or herself…”.*

(Participant 9)

In addition, the vulnerability of the caregiver him or herself, the absence of family being able to cope in terms of the care required by the vulnerable patient, and the existence of restricted financial family resources to provide care are other limitations that make home care difficult, as was outlined by our participants.

*“Sometimes the caregiver is as vulnerable as those who are cared for … many times you find that the family that provides the care is very fragile or vulnerable, they are elderly people with pathologies and limited resources who act as caregivers and who do everything possible to go on … but it’s very complicated”.*

(Participant 10)

#### 3.1.2. Subtheme 1.2: Perceived Barriers for the Involvement of Home Care Nurses in the Care

According to the point of view of our participants, one of the main barriers that hinder the home care of vulnerable patients is the lack of coordination between the different services involved in the care of vulnerable patients, as well as the existence of limited resources to cover their needs. This lack of coordination causes a delay in the arrival of the necessary resources for patients, directly affecting their state of health.

*“The assessment of the patient’s needs is multidisciplinary, this should not be done separately … but when all the patients have already been detected all their needs, and at a social level these resources to keep them in optimal conditions at home are lacking, this is often connected linked to a series of hospital admissions that entail a series of risks for the patient…”.*

(Participant 7)

On the other hand, the home care nurses in our study expressed their discomfort at the shortage of home care nurses available to carry out home care in the health services. This lack of human resources has a direct impact on the time available to carry out effective home care. In addition, our participants think that the bureaucratic tasks associated with nursing work also reduce the time spent with patients at home.

*“The care burden is too great. Home care is not something you do in 10 min, you have to see the people, their environment, which people have support, their resources … sometimes people in another house are waiting for me, and I forget to explain the risks of the home or preventive actions so that that person does not get worse … if there were more home care nurses, the quality of care we provide would greatly improve”.*

(Participant 12)

However, according to the opinion of our participants, there are factors inherent to the health professional that hinder home care. The provision of care outside the health center, in little known surroundings such as the patient’s home, caused insecurity in some home care nurses, making home care difficult. Due to this, a number of our participants acknowledged that they focus more on the techniques required by the patient than on the home approach.

*“In home visits we invade family privacy a bit, and there are issues that are complicated to deal with, such as hygiene and healthy conditions, etc. … it is easier to focus on what we know, which are basically the techniques …, but when we go to a home to carry out treatment or put in a urinary catheter, it is not a home visit”.*

(Participant 9)

The comprehensive care of the vulnerable patient not only includes the supply of their physical and social needs but also their emotional and spiritual needs. Our participants emphasized the importance of emotional support for the vulnerable patient. However, despite the fact that some of the participating home care nurses recognized the importance of the training and skills of the home care nurse for the comprehensive approach to vulnerable patients, they found emotional management difficult.

*“The home care nurse is well trained in home care, but many times there is a more emotional part … than plasters, how to brighten the patient’s day is more difficult …, this is often done at home, sometimes a joke cures more than a plaster that you put on their heel … ”.*

(Participant 11)

### 3.2. Theme 2: The Emotional Cost of Home Care

The daily care of vulnerable patients has a significant emotional impact on their caregivers. However, the home care of the vulnerable patient, given its complexity, also represents an emotional drain on the nurses. This topic shows the experiences of nurses regarding the emotional impact on caregivers as well as on themselves.

#### 3.2.1. Subtheme 2.1: Home Care Is Draining for Caregivers

Among the objectives of our participants is not only the care of the vulnerable patient but also care and attention to the caregiver him or herself, which was also one of their priorities. The home care nurses in our study reported that the care of the vulnerable patient, in addition to the physical impact, produces considerable emotional exhaustion in the caregiver, the majority of whom are forced to sacrifice their family, work and social life in order to devote themselves to the patient’s care. However, from the point of view of our participants, the role of the caregiver “is invisible to society”, which causes their physical and emotional needs to be socially masked.

*“The caregivers are people who do everything for nothing in return … it is a task that, from the outside, does not seem to exist … and it requires a lot of time, a lot of psychological wear and tear … and the caregivers are truly dedicated people, they not only dedicate effort and time but rather dedicate themselves entirely to the care of their family member, and this more important than preserving their own good health”.*

(Participant 8)

The role of home care nursing in the physical and mental well-being of the caregiver is essential according to our participants. Nevertheless, the total immersion of the caregivers in “their role of caring” resulted in the fact they were not really aware of their own needs and therefore did not accept the resources or support related to the delegation of care provided by the nurses. Our participants told us that the deterioration suffered by vulnerable patients causes caregivers to doubt their own abilities, which is why they consider health education and positive reinforcement of the caregiver to be important. According to the point of view of our participants, although the needs of the caregivers are infinite and the resources limited, the most outstanding needs are those of free time and emotional disconnection.

*“The caregiver is so immersed in the task of caring that they consider themselves essential in everything they do … they think that they are the only person who can give the correct care, and it is difficult to get out of this vicious circle … we home care nurses have to make them understand that “They are not Superman” and that the caregiver needs help and should ask for help … because they are people who normally do not attend to their own health problems or put them off … not voluntarily … but because they think that their main duty is to take care of their relative”.*

(Participant 3)

#### 3.2.2. Subtheme 2.2: The Impact of the Care on the Home Care Nurses

A large number of the participating nurses reported that the inability to solve the problems of a vulnerable patient and their caregiver at home affected their personal lives and generated, in addition to physical problems, feelings of frustration, grief and helplessness. Some home care nurses even felt vulnerable and that they were not sufficiently prepared to carry out home care. Other home care nurses experienced feelings of relief at changing health centers and being able to free themselves from the emotional burden of caring for certain vulnerable patients.

*“Although it’s work, it ends up affecting you, and there are situations that you don’t forget throughout your life … I remember having suffered from headaches, stomach pain, insomnia and having cried a lot with some patients … it’s clear that the suffering that we health professionals accumulate in our backpack is greater”.*

(Participant 4)

Despite the fact that some participants pointed out that the management of emotions is dependent on the personality of each home care nurse, the provision of care at home for pediatric patients or care at the end of life was emotionally shocking for our participants. For this reason, some home care nurses in this type of situation decided not to get involved in care as a defense mechanism to maintain their physical and emotional health. On the other hand, other home care nurses believed that distancing the vulnerable patient leads to the dehumanization of care.

*“You understand that as a professional you cannot collapse, there are situations that you knew would happen, and your mission is to alleviate, help, advise, collaborate or be there … in short it’s not my problem, it can’t make me suffer … but, if you become insensitive, you don’t do things with love, you dehumanize yourself … and care without the human element is not care…”.*

(Participant 9)

## 4. Discussion

The aim of this study has been to explore the experiences of home care nurses in the care of vulnerable patients. Home care nurses perceive a series of barriers to providing home care for this population. In addition, there is an emotional cost for both family caregivers and home care nurses. Communication and dialogue are essential to achieve the empowerment of patients and caregivers in self-care [17]. Home care nurses have to provide them with information they can understand and use, but the home care nurses encounter difficulties such as the sociocultural level of the patient and family [18,19]. This lack of communication prevents the creation of a close and trusting relationship necessary for patients to participate in decision-making regarding the care that is necessary at home, despite the efforts of the home care nurses [20].

One of the barriers that home care nurses encounter and that make home care difficult is the lack of social support resources in homes. Gender and socioeconomic inequality associated with care in the family environment can be found in other studies [21,22]. Both factors reduce access to formal resources by family members [23]. The woman continues to be responsible for home care [24,25]. However, the presence of female caregivers does not cancel the existence of this support deficit. In this sense, Rodríguez-Madrid et al. [26] concluded that the presence of women and family members does not correspond to good affectivity, nor does it imply that greater help is provided. Our research concluded that conflicts and the lack of coping on the part of family members make it difficult to provide care at home, and the home care nurses are defenders of the patient [27].

Coordination between social and health services is essential to care for vulnerable patients [13]. In this study an absence of socio-sanitary coordination on the part of the home care nurses is perceived. McGilton et al., [28], in a review on the care of chronic patients, propose a restructuring of social health systems in order to provide comprehensive assistance, continuity of care, and better access to services. In addition, the figure of a coordinator who leads the care that patients and family members should receive is essential [14]. They also point out that another variable that has made home care difficult is the shortage of home care nurses and the excessive bureaucratic tasks of nursing professionals, together with the insecurity caused by home care [29,30].

Caring for a vulnerable person puts an overload on the caregiver that results in their not being aware of their own well-being and even prevents them from developing their own abilities. On the other hand, the attention of home care nurses to this population involves an emotional drain [31]. They feel powerless because they cannot solve the problems of patients and their families, those they attend in their homes, and this generates an emotional discomfort that affects their personal, family and work lives [32]. Various studies have determined that home care nurses are exposed to scenarios of great emotional suffering when they face certain situations of vulnerability in the care they offer patients and their families [33]. This suffering generates syndromes such as compassion fatigue [34]. In the study by Pérez-García et al. [35] compassion fatigue produced a series of consequences at a personal and family level in home care nurses who even ended up with the intention or desire to leave the profession. In this study, distancing and job change were strategies used by home care nurses, despite believing that care was being dehumanized [36]. According to Angel and Vatne [37], attention to vulnerability lies in the home care nurse’s commitment to caring for the vulnerable patient, and if this does not occur, care for patients is not guaranteed. These facts denote the deficit of certain competences that must be present in the home care nurses, and this has been seen in the study. Skills such as empathy, compassion or resilience should be part of the training programs of health systems and organizations [38], and there are specific intervention programs based on mindfulness that focus on the cultivation or training of compassion [39]. In this way, home care nurses will be prepared to attend to situations with a high level of vulnerability and will be involved in the home care that they offer. In addition, it would be necessary to analyze if the social workers could help to improve the emotional discomfort that affects the personal, family and work lives.

Our findings highlight implications for policy. Social factors exert a clear influence on home care and should be accounted for in primary health care policy. The home care infrastructure for vulnerable patients is sometimes inadequate and a greater investment in material and human resources is necessary. A multilevel approach can help improve home care. Therefore, it is essential to develop strategies and programs adapted to the needs of vulnerable patients and their caregivers.

### Strengths and Limitations

The strength of this study is that the participants came from different health care centres, assuring representation of diverse points of views and the transferability of the findings. This study is not without limitations, however. Given the home care nurses’ demographic characteristics, the sample might not reflect the experience of home care nurses with less professional experience. Furthermore, the study has only collected nurses’ perceptions and opinions, and it would be recommended to include caregivers and vulnerable patients’ perspectives as well as other healthcare professionals and organizations involved in the care of vulnerable patients.

## 5. Conclusions

The findings of our study indicate that there are several factors which limit the involvement of home care nurses in the care of vulnerable patients. The sociocultural level of the patient and their family, the absence of a family and social support network, and the existence of family conflicts all complicate home care. The home care of a vulnerable person requires more material and human resources. Our findings emphasize the need for home care programs adapted to vulnerable patients. Home care is physically and emotionally exhausting for caregivers, but the total immersion in the role of the caregiver makes nursing care difficult for the caregiver. The difficulties in solving the needs of vulnerable patients provoke negative feelings in home care nurses. Further research is needed in order to understand the experiences of all persons and organizations involved in the home care of vulnerable populations.

## Figures and Tables

**Table 1:** Socio-demographic characteristics of participants.

Participant	Gender	Age	Marital Status	Years of Experience
1	Male	35	Unmarried	6
2	Male	56	Married	29
3	Female	40	Married	10
4	Male	38	Unmarried	5
5	Female	63	Married	20
6	Male	57	Cohabiting	35
7	Male	57	Married	29
8	Female	59	Divorced	33
9	Female	51	Divorced	21
10	Female	57	Unmarried	36
11	Female	59	Divorced	32
12	Female	54	Divorced	32
13	Male	44	Married	20
14	Female	31	Unmarried	5
15	Male	34	Unmarried	5

**Table 2:** Interview protocol.

Stage of the Interview	Subject	Content and Example of Questions
**Introduction**	My intention	I am a member of a research group which studies home care for vulnerable populations. Knowing their experiences could be useful for health care professionals and improving home care.
	Information and ethical issues	We need to record the conversation so that the research team can analyze the data. Only the research team will have access to the recordings. Participation is completely voluntary, and you can give up the study any time you wish. Your name and personal data will not be released.
	Consent	Signing of the corresponding acceptance document.
**Beginning**	Introductory question	As a home care nurse, what has been your experience with the care of vulnerable population?
**Development**	Conversation guide	Could you tell me what kind of difficulties you have encountered in caring for vulnerable patients and their caregivers? What are your goals in care for vulnerable patients? How do you involve patients and their caregivers in decision-making regarding their care and treatment? Could you tell me what are the most important needs of caregivers of vulnerable patients? What do you think about resources for vulnerable patients and their caregivers? Could you tell me how caring for the vulnerable patient has affected your personal life? And professionally?
**Closing**	Final question	Would you like to add something else?
	Appreciation	Thank you for your participation in the study. Your statement will be used for the research study. If you need anything, we remain at your disposal. You will receive the study when it is completed.

**Table 3:** Examples of coding strategy.

Quotation	Initial Code	Subtheme	Main Theme
*“A patient who needs help for self-care, connected to oxygen, who need medication and only has home help for two hours … really, that patient cannot handle their situation well, because they lack support”*	Lack of family and social support	The particularities of the patient and their home caregivers	Barriers to providing home care to vulnerable populations
*“there is no adequate space in homes and the care burden is too great. Home care is not something you do in 10 min, you have to see the people, their environment, which people have support, their resources…”*	Time and shortage of human resources	Perceived barriers for the involvement of home care nurses in the care	
*“the caregiver needs help and should ask for help … because they are people who normally do not attend to their own health problems or put them off … not voluntarily … but because they think that their main duty is to take care of their relative”*	Caregiver needs	Home care is draining for caregivers	The emotional cost of home care
*“Although it’s work, it ends up affecting you…I remember having suffered from headaches, stomach pain, insomnia and having cried a lot with some patients…”*	Negative effects	The impact of the care on home care nurses	

## Data Availability

Not applicable.
